# Whole-genome sequences of vaginal isolates *Lactobacillus crispatus* CECT30647 and *Lactobacillus gasseri* CECT30648

**DOI:** 10.1128/mra.00794-24

**Published:** 2024-09-30

**Authors:** Pol Huedo, Marta Perez, Eva Armengol, Antonio Del Casale, Ilenia Campedelli, Jordi Espadaler-Mazo

**Affiliations:** 1 R&D Department, AB-Biotics S.A. (Part of Kaneka Corporation), Barcelona, Spain; 2 Basic Sciences Department, Universitat Internacional de Catalunya, Barcelona, Spain; 3 Microbion SRL, Open Innovation Department, Verona, Italy; Loyola University Chicago, Chicago, Illinois, USA

**Keywords:** probiotics, biotechnology

## Abstract

We present the whole-genome sequences of *Lactobacillus crispatus* CECT30647 and *Lactobacillus gasseri* CECT30648, two vaginal isolates with probiotic potential.

## ANNOUNCEMENT

A healthy vaginal microbiota is typically dominated by *Lactobacillus crispatus, Lactobacillus gasseri*, and *Lactobacillus jensenii* species (1). Vaginal dysbiosis is associated to recurrent infections and reproductive complications (2). Genome sequencing of potential probiotics may contribute to characterize and select best candidates.

Strains *L. crispatus* CECT30647 and *L. gasseri* CECT30648 were isolated from vaginal swabs from two different Italian (Verona Province) healthy premenopausal donors, who gave informed, explicit consent. All procedures of isolation complied with the current laws of the country and adhered to the principles of the Declaration of Helsinki. Vaginal samples were seeded onto de Man, Rogosa, and Sharpe (MRS) agar plates and incubated at 37°C for 48 h in anaerobic jars. Bacterial species were firstly identified by species-specific primers (3) and verified through genomic Average Nucleotide Identity (ANI) analyses using JSpecies (4). Antibiotic susceptibility was performed following ISO10932:2010 (5) as recommended by EFSA (6). The strains were incorporated into the KANEKA Corp (Japan) collection (7) and deposited in the Spanish Collection of Type Cultures (CECT).

Isolated colonies were inoculated into 10 mL of MRS medium and cultured as indicated above. Cultures were centrifuged, and genomic DNA was extracted from pellets using the QIAamp DNA Blood Mini Kit (Qiagen, USA) following the manufacturer's instructions. Whole-genome sequences were obtained at Macrogen (Korea). Genome libraries were prepared by Nextera XT DNA Preparation Kit and sequenced by Illumina NovaSeq 6000 generating 2 × 150 bp paired-end reads. Trimmomatic (8) was used to remove adapter sequences and low-quality reads. *De novo* assembly was performed using SPAdes v3.13.0 (9). The assembled genomes were validated using BWA-MEM v0.7.17 aligner (10) and BUSCO analysis (11). Annotation was performed through NCBI Prokaryotic Genome Annotation Pipeline (PGAP) v6.7 (12). Presence of antimicrobial resistance genes was investigated in July 2024 through ResFinder v4.5.0 (13) and CARD (14), using complete genome sequences. Default parameters were used for all tools.

Genome sequencing of *L. crispatus* CECT30647 provided 33,133,782 total reads that were assembled in 154 contigs, comprising 2,130,379 bp, an N50 value of 26,707 bp, a 1,966 × coverage, and a 36.9% G + C content. PGAP annotation predicted 2,285 genes, including 2,100 protein-coding sequences and 68 tRNA, three 5S rRNA, two 16S rRNA, and one 23S rRNA sequences. The obtained genome displayed an ANI of 99.94% with *L. crispatus* PMC201 (GCF_018885325.1).

Genome sequencing of *L. gasseri* CECT30648 provided 15,207,330 total reads that were assembled in seven contigs, comprising 1,850,366 bp, an N50 value of 573,289 bp, a 1,020 × coverage, and a 34.8% G + C content. PGAP annotation predicted 1,783 genes, including 1,688 protein-coding sequences and 51 tRNA, three 5S rRNA, and one 23S rRNA sequences. The obtained genome displayed an ANI of 98.99% with *L. gasseri* UMB3077 (GCF_007786195.1).

*L. gasseri* CECT30648 showed phenotypic resistance to kanamycin and clindamycin, and *L. crispatus* CECT 30647 showed phenotypic resistance to kanamycin and streptomycin (Table 1). No kanamycin, clindamycin, or streptomycin resistance gene was detected in any genome. Only a putative and truncated *vanT* gene (30.8% identity; 50.6% coverage) was detected in the genome of *L. gasseri* CECT30648 through CARD analysis, which did not translate into vancomycin resistance. Future studies will determine if these strains can be considered probiotics.

**TABLE 1:** MIC of *L. gasseri* (*Lg*) CECT30648 and *L. crispatus* (*Lc*) CECT30647 to the antibiotics required by the EFSA^*a*^

Antibiotic	*Lg* CECT30648	*Lc* CECT30647	EFSA cut-off
Ampicillin	0.5 (S)	2 (S)	2
Vancomycin	2 (S)	1 (S)	2
Gentamicin	8 (S)	16 (S)	16
Kanamycin	128 (R)	128 (R)	16
Erythromycin	0.128 (S)	0.32 (S)	1
Clindamycin	8 (R)	1 (S)	4
Tetracycline	2 (S)	1 (S)	4
Chloramphenicol	4 (S)	2 (S)	4
Streptomycin	16 (S)	32(R)	16

^*a*^ S: susceptible; R: resistant.

## Data Availability

The whole-genome sequencing project for *L. crispatus* CECT30647 has been deposited at GenBank under the accession number JBFCOQ000000000. The raw sequencing data are available in the Sequence Read Archive (SRA) database under accession number SRR29792338. The associated BioProject accession number is PRJNA1132987. The whole-genome sequencing project for *L. gasseri* CECT30648 has been deposited at GenBank under the accession number JBFCOP000000000. The raw sequencing data are available in the Sequence Read Archive (SRA) database under accession number SRR29792339. The associated BioProject accession number is PRJNA1132975.
